# Idiopathic Recurrent Unilateral Pneumomastia: Diagnostic Challenges and Therapeutic Considerations With a Rare Case of Mammary Emphysema

**DOI:** 10.7759/cureus.100810

**Published:** 2026-01-05

**Authors:** Hajer Albalawi, Saleh M Alqarni, Wafaa A Alsati, Mohammed Y Alamassi, Ahmed G Elkhouly

**Affiliations:** 1 Thoracic Surgery, King Saud Medical City, Riyadh, SAU; 2 Radiology, King Abdulaziz Hospital, Jeddah, SAU; 3 Cardiothoracic Surgery, Tanta University, Tanta, EGY

**Keywords:** air within breast, breast parenchyma, breast swelling, case report, idiopathic breast emphysema, mammary emphysema, pneumomastia, rare breast disorder, subcutaneous emphysema, ultrasound-guided aspiration

## Abstract

Pneumomastia is a rare condition characterized by air within the breast tissue. It is typically caused by trauma, iatrogenic factors, or infections. We report a 22-year-old female patient with a 20-day history of progressive, painless unilateral left breast swelling and subcutaneous emphysema following a prior spontaneously resolved episode in the contralateral breast. Imaging revealed extensive extrapulmonary air within the left breast with subcutaneous emphysema. After exclusion of life-threatening causes, including pneumothorax and esophageal perforation, ultrasound-guided needle aspiration was performed, providing symptomatic relief but complicated by post-procedural infective mastitis. While most cases resolve spontaneously, invasive interventions should be guided by symptom severity. At the 12-month follow-up, the patient remained asymptomatic with no recurrence. A review of the literature indicates that idiopathic recurrent pneumomastia is an exceptionally uncommon presentation with scarce documented cases. This case highlights diagnostic challenges and outlines management strategies in idiopathic recurrent pneumomastia.

## Introduction

Pneumomastia, also known as mammary emphysema, is an exceptionally rare condition characterized by the presence of air within the subcutaneous tissue of the breast [1]. Its rarity is attributed to the anatomical barriers provided by the superficial mammary fascia, Cooper’s ligament, deeper fascia surrounding the retromammary space, and the pectoralis major muscle, which prevent air from entering the breast tissue [2]. However, in rare instances, air originating from the mediastinum or thoracic cavity may track into the breast subcutaneous tissue through natural anatomical pathways: via the axillary tail of Spence, which is the only area of breast tissue that penetrates the superficial fascia into the deep pectoral fascia, or via small projections of the mammary tissue that occasionally penetrate the deep pectoral fascia into the pectoralis major [3].

Reported etiologies include trauma [4], iatrogenic procedures [1,5-9], and infections [10]. Most cases follow recent instrumentation rather than occurring spontaneously [11], making idiopathic recurrent pneumomastia exceedingly rare and documented only in sporadic case reports.

Diagnosing pneumomastia can be challenging, necessitating a high index of suspicion and systematic exclusion of life-threatening conditions such as pneumothorax and esophageal injury [8]. Other important differentials include pneumomediastinum [3], iatrogenic causes [1,5-9], and necrotizing soft tissue infection [10]. Once serious etiologies are ruled out, most patients can be managed conservatively [8,11].

Herein, we report an unusual case of idiopathic recurrent pneumomastia in an adult female patient with a prior spontaneously resolved episode in the contralateral breast, complicated by post-aspiration infective mastitis.

## Case presentation

A 22-year-old female patient with no past surgical history or chronic medical conditions presented with a 20-day history of painless left breast swelling. The swelling had progressively increased in size, causing discomfort and sleep disruption without identifiable aggravating or relieving factors. She denied any associated dental, ear, nose and throat (ENT), respiratory, or systemic symptoms. A comprehensive history revealed no preceding events to account for the presentation, including trauma, recent procedures or surgeries, chest infections, inhalational exposures, or activities that increase intrathoracic pressure. Six months prior, she experienced a similar episode of right-sided breast swelling with mild subcutaneous emphysema, which resolved spontaneously without intervention. No precipitating factors were identified during that episode.

Upon presentation, the patient was conscious, alert, and oriented, appearing generally well. Vital signs were within normal limits: temperature, 36.8°C; heart rate, 97 beats per minute; blood pressure, 100/58 mmHg; and oxygen saturation, 98% on room air. Respiratory examination revealed a centrally located trachea with no thoracic scars or deformities. Percussion of the chest demonstrated normal resonance bilaterally, and auscultation revealed equal bilateral air entry with vesicular breath sounds.

On local examination, the neck was free of palpable lymphadenopathy, and no crepitus was noted. The left breast demonstrated diffuse, round, non-fluctuant, immobile, mildly tender, normothermic swelling with significant skin stretching and no overlying skin changes. Palpable subcutaneous emphysema with crepitus was present along the left lateral chest wall, extending posteriorly to the upper back. Examination of the right breast was unremarkable, and no bilateral axillary or supraclavicular lymphadenopathy was identified.

**Figure 1:**
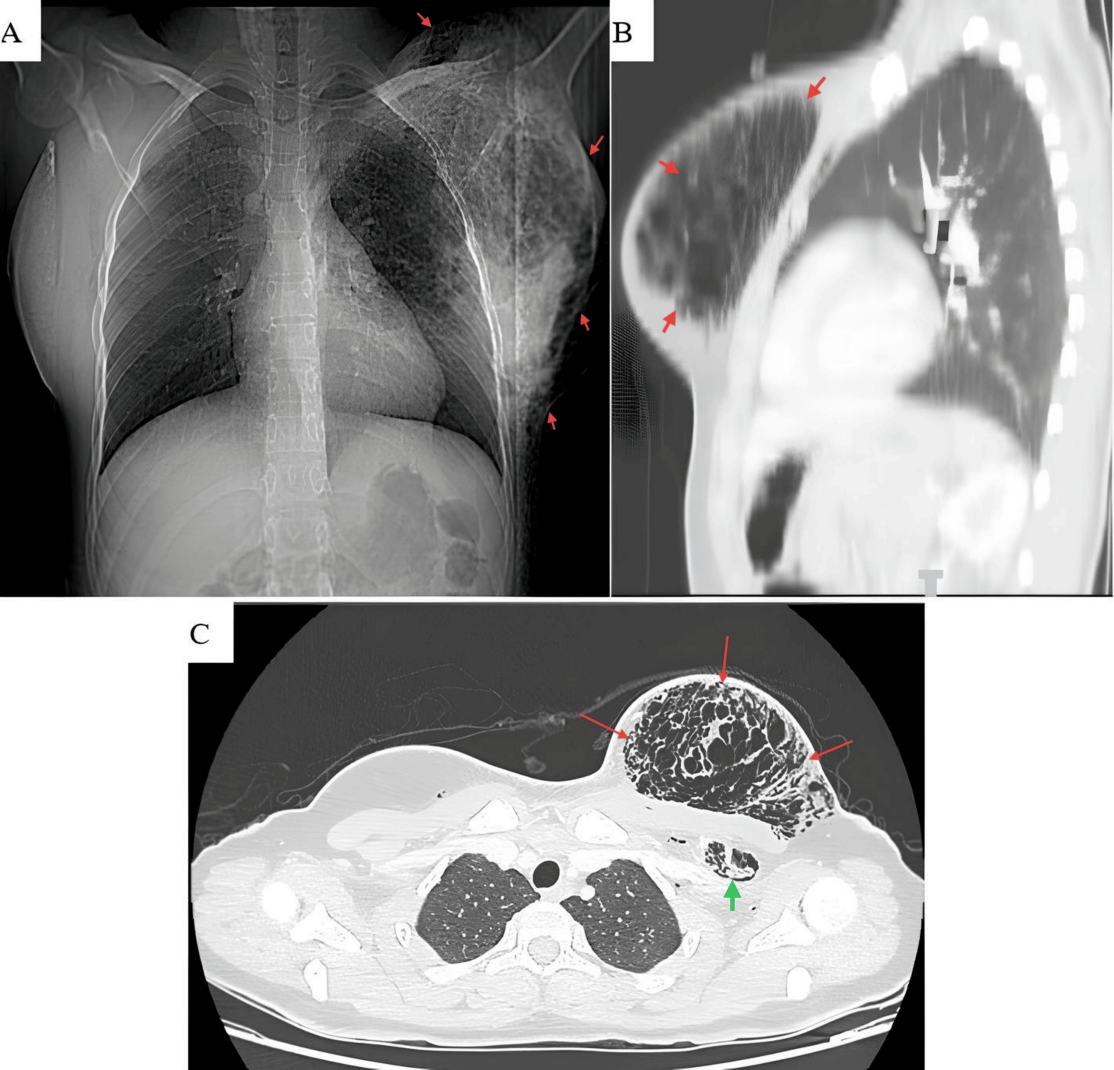
Imaging of left-sided pneumomastia (A) Posteroanterior chest X-ray revealing a large, well-circumscribed air collection along the left upper and lateral chest wall, replacing the majority of the left breast shadow (arrows), consistent with extrapulmonary air. (B) Sagittal contrast-enhanced chest computed tomography (lung window) showing a loculated air collection within the left breast (arrows). (C) Axial contrast-enhanced chest computed tomography (lung window) demonstrating an extensive, multiloculated air collection within the left breast, dissecting through the stromal connective tissue planes (red arrows). Air also tracks along the left anterior chest wall between the pectoralis major and minor muscles (green arrow). No evidence of pneumothorax or pneumomediastinum. No mediastinal or subcutaneous air tract is identified to suggest a broncho-cutaneous fistula.

All routine laboratory investigations were within normal limits. Imaging studies, including chest X-ray (CXR) and contrast-enhanced computed tomography (CECT), revealed an extensive, multiloculated extrapulmonary air collection within the left breast, accompanied by subcutaneous emphysema. No pneumothorax, pneumomediastinum, or radiological evidence of a broncho-cutaneous fistula was identified (Figure 1).

These findings were consistent with non-traumatic pneumomastia and associated subcutaneous emphysema.

Bilateral breast ultrasonography showed benign left axillary nodes with a preserved fatty hilum and echogenic air causing dirty shadowing throughout the left breast parenchyma, obscuring the underlying tissue. No solid or cystic masses were detected. The right breast was unremarkable.

**Figure 2:**
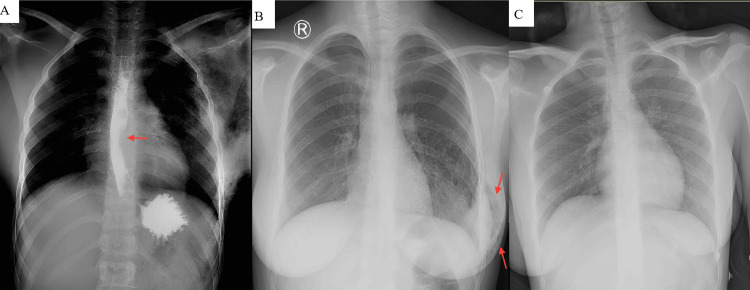
Imaging of left-sided pneumomastia with diagnostic evaluation, management, and follow-up (A) Frontal chest radiograph during fluoroscopic gastrografin swallow demonstrating free passage of contrast through the esophagus into the stomach (arrow), without evidence of contrast extravasation, fistula formation, abnormal stricture, or filling defect. (B) Posteroanterior chest X-ray following ultrasound-guided needle aspiration reveals marked reduction of left-sided pneumomastia, restoration of normal soft-tissue density, and improvement of subcutaneous emphysema, with a trace remaining (arrows). (C) Twelve-month follow-up chest X-ray confirms complete resolution of the left pneumomastia, with no evidence of recurrence in the breast or subcutaneous tissue.

Esophageal perforation was ruled out using a fluoroscopic gastrographic study, showing free passage of contrast without leakage (Figure 2A).

To further explore potential air leakage, flexible nasopharyngolaryngoscopy and fiberoptic bronchoscopy was performed and revealed no abnormalities. 

Ultrasound-guided needle aspiration of the left breast was performed due to significant discomfort with associated tension and marked cosmetic concern. Management options, including observation for spontaneous resolution versus needle aspiration, were discussed, and the patient opted for the procedure to relieve her symptoms. The intervention resulted in a notable reduction in pneumomastia size, alleviating tension and discomfort (Figure 2B), with only mild tenderness persisting at the aspiration sites and no skin changes.

Despite extensive radiological evaluation and procedural interventions, the exact source of the pneumomastia remained undetermined. However, given the resolution of symptoms, the patient was discharged after four days in good condition with plans for outpatient follow-up.

Seven days later, the patient returned to the emergency department with new-onset localized left breast pain, accompanied by chills, myalgias, and malaise. On clinical examination, the left breast showed tenderness, erythema, and warmth at the previous aspiration sites, with no evidence of pneumomastia or subcutaneous emphysema. Vital signs included a low-grade fever (38 °C; reference range: 36.1-37.2 °C) and mild tachycardia (110 bpm; reference range: 60-100). Laboratory evaluation revealed leukocytosis (white blood cell count (WBC) 13 × 10⁹/L; reference range: 4-11 × 10⁹/L). Based on the clinical presentation and laboratory findings, a diagnosis of post-aspiration infective mastitis was made. The patient was hospitalized and started on intravenous amoxicillin-clavulanate and analgesics. Given the mild presentation, absence of abscess formation, and rapid clinical response, cultures were not obtained. After 72 hours of improvement, she was discharged in stable condition on a 10-day course of oral amoxicillin-clavulanate, with a scheduled outpatient follow-up.

**Figure 3:**
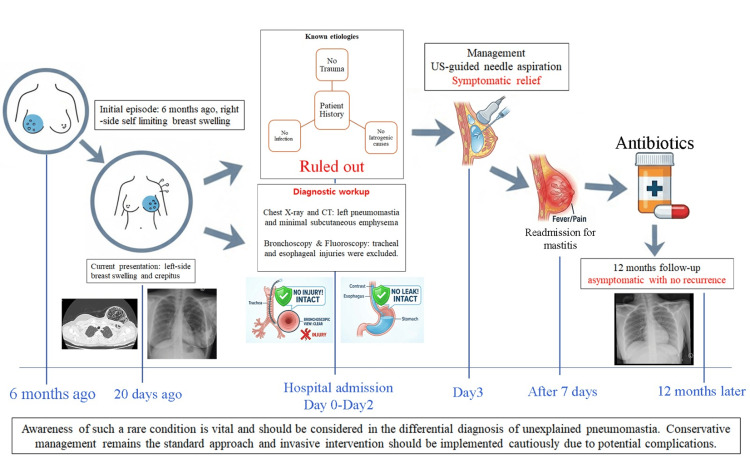
Clinical timeline of the patient with idiopathic pneumomastia. Timeline illustrating symptom progression, diagnostic evaluation, interventions, and 12-month follow-up; US: Ultrasound. Image credit: Created by Dr. Elkhouly A using Photoshop (Adobe Inc., California, US).

At the six- and 12-month follow-up visits, the patient remained asymptomatic, and chest radiography confirmed no recurrence of pneumomastia or subcutaneous emphysema (Figure 2C). She was counselled to avoid activities that increased intrathoracic pressure, including strenuous exercise, forceful straining, vomiting, or vigorous coughing, as well as inhaled stimuli likely to provoke airway strain. The patient was informed about the possibility of recurrence, reassured regarding the favorable, self-limiting course, and advised that conservative management is expected. She was instructed to promptly seek medical evaluation for any new chest wall swelling, crepitus, pain, or other unusual symptoms. Further imaging was reserved for new or concerning findings (Figure 3).

## Discussion

Pneumomastia is a rare condition often linked to identifiable etiologies [11], broadly classified into traumatic [4], iatrogenic [1,5-9], and infectious causes [10]. Among the trauma-related causes, pneumomediastinum may arise from airway injury or increased alveolar pressure (vigorous coughing, Valsalva maneuvers, or asthma exacerbations), allowing air to dissect along tissue planes into the breast [3]. More directly, trauma-related pneumomastia may occur due to chest trauma, rib fractures, or post-cardiopulmonary resuscitation (CPR) [4].

Iatrogenic causes include breast augmentation surgeries that permit air entry due to direct manipulation of breast tissue during procedures [5]. Another cause is thoracostomy tube placement in patients with breast implants, which creates a valve-like defect in the implant capsule, allowing air to accumulate within the breast tissue [6]. Other, rarer mechanisms of pneumomastia include insufflated gas migration during laparoscopic surgeries [1], inadvertent air dissection following intravenous catheter placement [7], and bronchopleural fistulas following redo cardiothoracic surgeries [8] and Vibration Amplification of Sound Energy at Resonance (VASER)-assisted liposuction [9]. Collectively, the most reported cases of pneumomastia are associated with prior instrumentation or surgical procedures, consistent with previous studies [11]. In contrast, our patient had no identifiable cause, reinforcing the idiopathic nature of the condition.

Furthermore, infectious etiologies, particularly anaerobic infections which produce gas within the breast tissue, are typically accompanied by systemic symptoms and localized signs of infection [10].

Pneumomastia usually presents unilaterally, while bilateral cases are rare and typically linked to extensive air dissection from mediastinal sources [3]. Clinical manifestations depend on the underlying cause, but common symptoms include breast swelling, mild tenderness, and subcutaneous emphysema with palpable crepitations [7]. Although it is often asymptomatic [11], our patient uniquely presented with two distinct episodes of tense breast swelling, initially on the right side and six months later on the left, each occurring in the absence of systemic symptoms along with normal vital signs and inflammatory markers. This case demonstrates that recurrence, though uncommon, can occur.

Here, a thorough diagnostic workup was performed to exclude potential causes of pneumomastia. The differential diagnosis included spontaneous pneumothorax, pneumomediastinum, bronchopleural fistula, cystic or neoplastic breast lesions, esophageal perforation, and tracheobronchial injury. These were systematically excluded based on the patient’s history, clinical assessment, imaging, and airway endoscopy, all of which were unremarkable. Despite extensive investigations, no definitive cause was identified, underscoring the necessity of a comprehensive diagnostic evaluation to exclude all serious underlying pathologies before establishing a diagnosis of idiopathic pneumomastia.

Management of pneumomastia is typically conservative, focusing on symptomatic relief, addressing underlying causes when present, and close monitoring for complications [11]. Given the self-limiting nature of most cases, invasive interventions should be tailored to the patient’s clinical condition. In our case, ultrasound-guided needle aspiration was performed due to significant discomfort with tension and cosmetic concern. The patient opted for the procedure after discussion of management options, achieving immediate symptomatic improvement. However, the procedure was complicated by the development of post-aspiration infective mastitis. This aligns with prior recommendations that invasive procedures, such as needle aspiration or drainage, should generally be avoided to mitigate complications like open pneumothorax or bronchocutaneous fistula [8]. 

At the six- and 12-month follow-up, the patient remained asymptomatic with no clinical or radiologic evidence of recurrence, highlighting the favorable prognosis of idiopathic pneumomastia in this case despite the need for invasive management.

## Conclusions

This case emphasizes the necessity of a comprehensive, systematic evaluation to exclude all potential causes, including trauma, infection, and iatrogenic factors, prior to establishing a diagnosis of idiopathic pneumomastia.

Life-threatening thoracic conditions, such as pneumothorax and esophageal perforation, must be excluded before initiating conservative management, which remains the mainstay of treatment due to the condition’s typically self-limiting course. Invasive interventions, such as ultrasound-guided aspiration, should be reserved for carefully selected patients with significant or persistent symptoms. While it provides symptomatic relief, the procedure carries a risk of post-procedural complications, highlighting the importance of weighing potential benefits against risks.

Idiopathic recurrent unilateral pneumomastia is exceptionally rare and remains poorly characterized due to the limited number of reported cases. Clinicians should consider idiopathic pneumomastia in the differential diagnosis of unexplained breast swelling with subcutaneous emphysema, and close follow-up is recommended to monitor for recurrence or emerging pathology.
